# Implementing the general capabilities in New South Wales government primary schools

**DOI:** 10.1007/s41297-022-00169-5

**Published:** 2022-07-13

**Authors:** Don Carter, John Buchanan

**Affiliations:** School of International Studies and Education, University of Technology Sydney, Sydney, NSW Australia

**Keywords:** General capabilities, Australian Curriculum, Primary schools, Curriculum implementation

## Abstract

This paper reports on the findings of a research project which investigated how a sample of New South Wales government primary schools understand and implement the Australian Curriculum’s general capabilities. The project sought to identify specific factors which facilitated or hindered the degree to which primary school teachers implemented the GCs in their classrooms. Data were collected and analysed in the period leading up to the current COVID-19 pandemic and as such, do not address current contextual factors at play in schools such as remote teaching and learning. The project’s mixed method approach employed an online survey which attracted responses from 185 primary teachers and included an invitation to provide brief written responses. Further data were assembled through 36 interviews undertaken with teachers in 12 primary schools in both metropolitan and rural NSW. Analysis of the interview data was undertaken by using Biesta’s (2010) three functions of education — qualification, socialisation and subjectification — as an interpretive lens. This enabled the researchers to identify, categorise and accord meaning to participant responses and consequently draw conclusions. The analysis of both the online survey and the teacher interviews revealed four main findings and shed light on individual teacher commitment to the general capabilities and associated classroom implementation issues.

## Introduction

The transformative role of education through its focus on issues such as justice/injustice, democracy, citizenship and sustainability has long been recognised as important (Apple, 2009; Beckett, 2012; Dewey, 1938). The inclusion of the general capabilities (GCs) and cross-curriculum priorities in the Australian Curriculum (AC) have also been hailed as significant due to a similar focus (Akshir and Kadiris, 2018; Grainger et al., 2019). This paper investigates New South Wales (NSW) primary school teachers’ understanding and classroom implementation of the Australian Curriculum’s general capabilities. One-hundred eighty-five primary school teachers responded to an online survey, 36 teachers from 12 NSW primary schools being interviewed. The study took place in the lead-up to the current COVID-19 pandemic. Accordingly, the data do not refer to aspects of the pandemic and this paper makes no recommendations related to school-based education in a pandemic context. However, the data gleaned from the project provide insights into the degree to which NSW primary teachers understand and value the GCs and how these capabilities have been implemented in classrooms. The data also align with Ben-Peretz’s (1990) assertion that curriculum implementation is more than the “faithful transmission of developers’ intentions” (Deng, 2011, p. 540) and in fact entails “curriculum potential” (Shulman, 1990, p. 7) where unintended but rich student learning outcomes are a feature of teachers’ “diverse interpretations and uses of curriculum materials” (Deng, 2011, p. 540). The findings are relevant to curriculum and school-based teaching/learning program designers, and curriculum researchers.

## Background

Curriculum studies is an area of ongoing contestation and dispute (Deng, 2011; Henderson, 2020; Savage & O’Connor, 2015). Central to this is the worth and value of knowledge to be included or excluded in a curriculum (Henderson, 2020; Kennedy, 2019) or what students “ought to learn” and what they “ought to become” (Henderson, 2020, p. 205). Various debates characterise curriculum as “‘planned’ or ‘enacted’” (Yates, 2018, p. 138) and identify a tension between curriculum that equips students with future-oriented skills and curriculum that develops young people’s knowledge and understanding of their cultural heritage and national history (Henderson, 2020, p. 203). Curriculum debates are “debates about a nation’s soul. About its values. About its beliefs” (Kennedy, 2019, p. 121).

In Australia, a “constitutional division of powers in education” (Harris-Hart, 2010, p. 296) assigns control to state governments; however, in 2008, all Australian states and territories agreed in principle with the development of an Australian Curriculum to “deliver an equitable, quality education for all young Australians” (Australian Curriculum Asessment Reporting Authority, n.d.). A curriculum that “meets the political and management desire for greater uniformity, for common measures, for easier transfer” (Yates et al., 2011, p. 324) underpinned the view of its advocates, whilst opponents argued that a national curriculum would, amongst other things, “remove scope for competition, comparison and diversity” (Drabsch, 2013, p. 20).

Over the following 7 years, the agency charged with developing this project, the Australian Curriculum Assessment and Reporting Authority (ACARA), developed content in English, Mathematics, Science, History, Geography, Personal and Physical Education, Technologies and the Arts.

The AC includes seven general capabilities: literacy, numeracy, ethical understanding, information and communication technology capability, critical and creative thinking, intercultural understanding and personal and social capability. The GCs set out to develop in young people:

a range of generic and employability skills that have particular application to the world of work and further education and training, such as planning and organising, the ability to think flexibly, to communicate well and to work in teams. Young people also need to develop the capacity to think creatively, innovate, solve problems and engage with new disciplines (Australian Curriculum Assessment Reporting Authority, 2012, p. 14).

Also developed were the cross-curriculum priorities of Aboriginal and Torres Strait Islander histories and cultures, Asia and Australia’s engagement with Asia and Sustainability. These priorities were created to enrich study of the learning areas and to encourage “conversations between learning areas and between students, teachers and the wider community” (Australian Curriculum Assessment Reporting Authority, 2012, p. 18).

However, the development and implementation of Australia’s national curriculum was not straightforward; curriculum reform in Australia has continuously been “protracted and complex” (Savage, 2016, p. 835), underscoring the “always contentious” (Brennan, 2011, p. 259) nature of curriculum. Previously, when the prospect of a national curriculum was floated, states and territories activated a rear-guard action which “jealously guarded their curriculum sovereignty, overtly or passively resisting attempts to engineer national approaches” (Reid, 2005, p. 15 quoted in Hughes, 2019). Reid (2019) also notes the longstanding tension between the “nation-building aspirations” (p. 199) of the Commonwealth government and the “constitutional responsibility” (p. 199) of the States, providing the backdrop for a complex blend of globalisation, various educational ideologies and practices and “curriculum gatekeepers” (p. 199). The issue of “States’ rights sentiments” (p. 203) continued to feature during the AC’s development.

## The AC and the states/territories

Adopting and implementing a national curriculum was further complicated by several factors. First, curriculum development across Australian States and Territories is the responsibility of each respective government. In NSW, for example, the *NSW Education Act* (1990) requires that curriculum implemented in schools must be developed by the New South Wales Education Standards Authority (Education Standards Authority Act, 2013, Sect. 3) to be “implemented within the NSW legislative framework” (Board of Studies, 2010, p. 3). In Victoria, the Victorian Curriculum and Assessment Authority is similarly responsible for curriculum development (Sect. 2.5, Education and Training Reform Act, 2006). This may explain why both NSW and Victoria “asserted their independence” (Reid, 2019, p. 203) by retaining their own respective and distinctive curriculum features whilst “aligning it with the Australian Curriculum” (p. 203), and more generally, why the States did not uniformly approach AC implementation (Akshir and Kadiris, 2018, p. 536).

A second factor also influenced NSW’s national curriculum approach. Hughes (2019) labels this factor NSW’s “distinctive curriculum style” (p. 147), encompassing three principal characteristics: “academic knowledge”, “competitive assessment” and “conventional subject matter disciplines” (p. 147), underscoring NSW’s long-established “reputation for developing and implementing high-quality curriculum” (Board of Studies, 2010, p. 2). The NSW response to the draft AC derived from broad stakeholder feedback, criticising numerous aspects including the absence of an overarching curriculum framework, time allocations for subjects, the amount of content and the absence of a clear continuum of learning aligned with the Early Years Framework (Board of Studies, 2010, p. 5). Specifically, the Board’s response sought to “clarify the exact role” of the GCs and their “relationship to the subject content” (Board of Studies, 2010, p. 16). Citing the Literacy and Numeracy GCs, the response asserted that these are “different in nature” (p. 16) due to their centrality to the formative years of schooling and should therefore be “treated differently” (p. 16) through provision of a “specific scope and sequence of learning” (p. 16), whereas the other GCs such as “ethical understanding” and “intercultural understanding” can be seen as “dispositions, characteristics or emphases” (p. 17) and are developed through the content areas.

However, the Board of Studies’ response (2010) clearly indicated its commitment to “working with all other states and territories to achieve a high quality core Australian curriculum” (Board of Studies, 2010, p. 2); thus, NSW embraced an “adopt and adapt” process (Hughes, 2019, p. 153) meaning that if NSW had no syllabus prior to the Australian Curriculum, it would “adopt the national program” (p. 153), whilst it would “adapt” the content into existing NSW programs (p. 153). NSW had existing syllabuses in English, Mathematics, Science and History, into which AC content was integrated in NSW Kindergarten-Year 10 syllabuses released between 2014 and 2016.

## The general capabilities in Australian states/territories

Implementing the general capabilities in the respective Australian States and Territories was undertaken in different ways (Gilbert, 2019, p. 173). In NSW, the GCs were embedded “where appropriate within NSW content” (Board of Studies, Teaching and Educational Standards, 2014, p. 5), with the GCs identifiable through an assigned code aligned with relevant content descriptions. The GCs were also identified as “useful reference points for thematic programming” (p. 5) as well as “illustrative material to assist in contextualising the content learning” (p. 5). Queensland adopted a similar approach, with statements of learning distinguishing the capabilities of literacy, numeracy and ICT as those which “support students to be successful learners” and the remaining capabilities as those which “develop ways of being, behaving and learning to live with others” (Queensland Curriculum and Assessment Authority, 2015).

The Victorian Curriculum and Assessment Authority (VCAA) advised that the capabilities of literacy, numeracy and ICT can be embedded into relevant parts of the curriculum (VCAA, 2015, p. 13) and are distinct from the remaining GCs which are “constituted by a discrete set of knowledge skills that are not fully incorporated in any one of the learning areas” (p. 44) to be presented in this curriculum as “distinct areas of learning” (p. 44). In Western Australia, teachers are expected to both teach the GCs and assess student achievement accordingly, “to the extent that they are incorporated within each learning area” (School Curriculum and Standards Authority, 2020). In South Australia, Tasmania, the Northern Territory and the Australian Capital Territory, the Australian Curriculum is implemented in schools, rather than through state-developed curricula incorporating the GCs.

## The present study

This project sought to investigate how NSW primary schoolteachers understand the GCs and to ascertain the extent to which the GCs are implemented in their classrooms. To enlist NSW primary schools for data collection, the chief investigator emailed 85 NSW school principals. Additionally, school principals in the Riverina, in rural south-west NSW and the mid-north coast were invited to participate in an online survey and face-to-face interviews, as were their teaching staff. Consequently, 185 teachers completed the online survey and 12 schools volunteered to participate, with 36 face-to-face teacher interviews taking place.

The online survey employed non‐random sampling as the data compilation method and Likert scales to “build in a degree of sensitivity and differentiation of response while still generating numbers” (Cohen et al., 2011, p. 325). The survey questions targeted three key issues:

1. How participants perceive their own understanding of the GCs;
2. Whether participants implement the GCs in the classroom, and if so, how this is done;
3. If individual schools promote the integration of the GCs in their teaching and learning programs.

The survey’s open‐ended questions sought to catch the “authenticity, richness, depth of response, honesty and candour” and “gems of information”, the hallmarks of qualitative data (Cohen et al., 2011, p. 266). These word‐based data were analysed and interpreted using an inductive, iterative approach. Although such an approach might limit the generalisability of the findings, teacher responses reported here contribute to a growing body of research that emphasises the voices of professionals in a wider educational, political and socio‐media landscape that often devalues and marginalises their professionalism, expertise and judgements.

The project also adopted semi-structured interviews for their “affinity with qualitative, quantitative and mixed-method research” (McIntosh & Morse, 2015, p. 1) where a sense of empathy between researcher and interviewee enables them to “collaborate to produce knowledge” (p. 4). Semi-structured interviews also elicit open responses that “enable lines of conversation to be developed in situ in ways that could not have been anticipated when the interview schedule was being planned” (Brown & Danaher, 2019, p. 77).

The project utilised thematic analysis, a “process of identifying patterns or themes within qualitative data” (Maguire & Delahunt, 2017, p. 3352). Since the early twentieth century, “thematic analysis” has referred to, *inter alia*, data analysis techniques in the social sciences (Terry et al., 2017, p. 1). Careful data interpretation is required when “identifying, analysing and reporting patterns (themes)” (Braun & Clarke, 2006, p. 79) providing a “flexible and useful research tool, which can potentially provide a rich and detailed, yet complex, account of data” (p. 78).

Analysis was undertaken to discern two thematic levels (Braun & Clarke, 2006), the “semantic” and “latent” (p. 84) in the interview data. Semantic themes operate “within the explicit or surface meanings of the data and the analyst is not seeking anything beyond what a participant has said or what has been written” (p. 84), whilst the latent level investigates deeper, beyond what has been uttered and “starts to identify or examine the underlying ideas, assumptions and conceptualisations – and ideologies—that are theorised as shaping or informing the semantic content of the data” (p. 84). The research team sought to identify participants’ relevant educational practices whilst also uncovering underlying attitudes and beliefs that inform and direct these practices.

Code-generation in the semantic analysis was undertaken through the identification of specific classroom approaches offered in participant responses. Specific words or phrases were developed to signal the essence and meaning of participant responses, such as “textual/literary study” and “drama”.

To analyse the latent data themes, Biesta’s (2010) three functions of education — *qualification*, *socialisation* and *subjectification* — were utilised as an interpretive lens to illuminate participants’ educational priorities not necessarily readily visible or articulated in full. The application of these functions provided insight into the “multidimensionality of educational purpose” (Biesta, 2013, p. 128).

## Participants’ background

The “Introduction” section required participants to provide demographic information. This is summarised in Table 1, below.

**Table 1:** Survey participant demographics

Category	Numbers (and percentages)
Female	157 (85.3%)
Male	27 (14.7%)
English as a first language	176 (95.7%)
Other language backgrounds	8 (4.3%) (see further details below)
Sydney metropolitan location	92 (50%)
Regional centre	45 (24.5%)
Rural location	42 (22.8%)
Remote location	5 (2.7%)
1–5-year experience	33 (17.4%)
6–10-year experience	45 (24.5%)
11–20-year experience	52 (28.3%)
Over 20-year experience	55 (29.9%)
Classroom teachers	95 (51.9%)
Executive/leadership positions	47 (25.4%)
Other positions (librarians, support staff)	16 (8.8%)
Executive/leadership positions	47 (25.4%)
Aboriginal Torres Strait Island background	7 (3.8%)

The following were identified as first languages: Filipino (1), Greek (2), Italian (1) and Vietnamese (2). In the 36 face-to-face teacher interviews across 12 schools, grades and geographic locations are outlined in Table 2, below.

**Table 2:** Interviewee grades and location

Category	Numbers (and percentages)
Early stage 1 (kindergarten)	6
Stage 1 (years 1 and 2)	8
Stage 2 (years 3 and 4)	5
Stage 3 (years 5 and 6)	13
South-west Sydney	2
Mid North Coast	1
Riverina (south-West NSW)	3
Queanbeyan (Southern Tablelands)	6
Female	30
Male	6

In addition, one interviewee identified as a principal; two identified as support teachers; and another, a school librarian. A breakdown of experience and grades is presented in Table 3, below.

**Table 3:** Interviewee experience and grades taught

Early career (0–5 years)	Mid-career (6–10 years)	Later career (10 years or more)
5 (early stage 1)	7 (stage 3)	1 (principal)
5 (stage 1)	2 (stage 2)	1 (librarian)
3 (stage 2)	1 (early stage 1)	2 (support teachers)
	3 (stage 1)	6 (stage 3)

## Online survey

The survey comprised four sections, the first comprising a rationale for the survey and its overall purposes. The “Background” section requested participants’ school and teaching experience information. The “The general capabilities ” section elicited participants’ knowledge and understanding of the general capabilities, whilst the final section elicited participants’ classroom priorities, including the degree to which the GCs were prioritised.

## Survey results

Participants were asked to rate statements on their understanding of the GCs according to a five‐point Likert Scale from “strongly agree” to “strongly disagree”. The results are outlined in Table 4, below.

**Table 4:** Responses to general capability statements

Statement	*n* =	Strongly agree	Agree	Unsure	Disagree	Strongly disagree
I understand the GCs	180	21 (11.7%)	75 (41.7%)	48 (26.7%)	28 (15.6)	10 (5.5%)
I consider the GCs important in the curriculum	182	39 (21.4%)	72 (39.6%)	50 (27.5%)	10 (5.5%)	11 (6.6%)
My school prioritises teaching of the GCs	183	14 (7.7%)	43 (23.8%)	64 (35.4%)	37 (20.4%)	23 (12.7%)

From the above table, a mismatch emerges between ascription of the importance of the GCs, and an understanding and implementation thereof. The participants were in strongest agreement about the importance of the GCs. They were less confident about their knowledge of them, and less in agreement again about school prioritisation. Participants were also asked to respond with “Yes”, “No” or “Occasionally” to the following statement: *I use specific teaching strategies/activities to implement the general capabilities*. Responses are tabulated below (Table 5).

**Table 5:** Use of specific GC teaching strategies/activities (*n* = 184)

Statement	Yes	Occasionally	No
I use specific strategies to implement the GCs	55 (29.1%)	67 (36.4%)	62 (33.7%)

From the above table it can be seen that one in three respondents is not using GC-related strategies at all, whilst more than a third of the remainder only uses them occasionally, amassing a total of 70.9 percent of non- or occasional users.

## Discussion: survey results

As indicated above, the responses to *2.1 — I understand the general capabilities* indicate that only a small majority (53.4%) consider that they understand the GCs, with 15.6% stating they lack understanding of the GCs. Written responses to this statement reveal numerous factors impacting the extent of their understanding of the GCs. Written responses from the 26.7% who are “unsure” and those indicating they do not understand contend that a lack of professional development impedes their GC understanding. This was evident in the responses of 28 (15.6%) participants who identified a lack of understanding of the “ethical understanding” and “intercultural understanding”. Furthermore, 15 (8.1%) participants commented that the inclusion of the GCs in classroom programming on top of syllabus outcomes, content and literacy/numeracy strategies would be onerous and deflect attention from these priorities.

The responses to *2.2 — I consider the general capabilities to be an important element of the curriculum* indicated that 11 participants (6%) disagreed or strongly disagreed. Rather than disagreeing with the principles underpinning the GCs, participants cited other factors impinging on their time and energy to prioritise the GCs. These included preparation and responses to NAPLAN (National Assessment Program Literacy and Numeracy) test results, the behaviour management of students, programming requirements of syllabuses which a number of participants labelled as “content heavy”, accountability and administration duties and extra-curricular obligations.

Responses to *2.3 — My school prioritises the teaching of the general capabilities* indicate that 33.9% of schools prioritise the teaching of the GCs, whilst 26.7% signalled that their schools do not, and 39.3% were unsure. Of those whose schools prioritise this, 26 stated that this was done informally, rather than through the formal programming in teaching/learning programs.

### Teaching/learning strategies used to implement the general capabilities

It is worth noting here that the NSW Curriculum requires teachers to base their teaching/learning programs on syllabus outcomes and assess student achievement of those outcomes across a 2-year learning stage. Teachers are not required to specifically program the GCs or report on student achievement therein. The project sought to understand if teachers did in fact deliberately integrate the GCs into their teaching/learning programs. Participants were invited to respond Yes, No or Occasionally to the statement “I use specific strategies/activities to implement the general capabilities” (see Table 4, above).

Those who responded Yes or Occasionally were invited to provide a short-answer response to specify their strategies and activities. Of these, 13 indicated that they “embed” the GCs into their classroom programming, through textual study (picture books, big books and chapter books) and discussion to strengthen GCs such as “intercultural understanding”, whilst six participants used project-based learning and inquiry-based learning with one asserting that such an approach provides “flexible pedagogy” allowing for student differentiation more readily than through conventional direct instruction. In addition, three participants emphasised the use of ICT in the classroom and reported that various software programs allow them to integrate the GCs into their classroom teaching. These participants indicated that they regularly use the NSW Department of Education’s *Virtual manipulatives*, allowing students to “engage with concrete materials on digital devices to support their understanding of abstract ideas” (NSW Department of Education, n.d.).

Two participants reported that they use drama and roleplay to target aspects of the GCs, particularly “intercultural understanding”. Both indicated high proportions of culturally and linguistically diverse students and argued that drama activities were effective in opening spaces for discussion of issues related to cultural practices, traditions and languages. Of note is a significant absence of comments on other curriculum key learning areas, for example visual arts, history, geography and physical education. This might provide an area for future research.

### Interviews

The 36 semi-structured interviews sought to “allow the examiner to dig deep into the experiences and/or knowledge of the participants in order to gain maximum data” (Turner, 2010, p. 757). To this end, the questions were designed as neutral, clearly worded and sufficiently open-ended, allowing respondents to choose their own terminology (McNamara, 2009). Specific questions targeted participant familiarity with the GCs, and if and how they might be implemented into participants’ teaching programs. The interviews were undertaken to ascertain the degree to which participants were familiar with the GCs and to determine the role the GCs might (or might not) play in their classroom teaching.

### Results

Of the 36 interviewees, three conceded unfamiliarity with the GCs, and all called for further professional learning on the GCs. The other 33 interviewees, who demonstrated specific and detailed knowledge of the GCs, cited instances of GC inclusion in classroom interactions and teaching. What was apparent in these responses was the commitment to the principles and ideas underpinning the GCs. All participants agreed that the GCs were an important curriculum component, the following comment being typical: “(the GCs) are front and centre of planning” (M/LC^1^). The GC “personal and social capability” (PSC) was identified by all participants as being central to their work as teachers. One participant argued that this GC is “our core business” [1] (F/ECa) whilst another noted that PSC enabled “our kids to understand themselves as learners” (F/LC).

Ten participants indicated that they cover the GCs informally. Whilst they were aware of the GCs, they expressed the view that they as a matter of course in their teaching cover the principles of the GCs in classroom units of work, such as textual study, rather than through formal programming. One specific example was the study of the *Once* series by Morris Gleitzman. This participant discussed how she initiated “ethical and cultural conversations” (F/MCa) through the storyline and characters in this novel set in Nazi-occupied Poland in 1942. She further explained that each day offered numerous opportunities to address the GCs, albeit informally.

Eleven participants recounted a more formal approach to the GCs. This entailed the programming of the GCs into teaching/learning programs. For all participants, this required the inclusion of specific GCs in the school’s teaching/learning programming formats which had been revised to accommodate this feature. Usually a simple tick/check was required to indicate which GCs were to be covered in specific units of work, and a review undertaken at the conclusion of each school term and year ensured what each school considered to be a relevant and comprehensive coverage of the GCs.

The responses of two participants were distinctive. One (M/MC) explained that the GCs were incorporated into school reports for parents. Student progress in relation to each GC was depicted in the reports as “working at” or “working beyond” (M/MC). Additionally, students were invited to self-assess their engagement with the GCs by undertaking the same process as the class teacher. He added, “You get a lot of kids that are quite accurate. I’d agree with how they rate themselves. They’re pretty honest with themselves” (M/MC). This self-assessment is also forwarded to parents/caregivers.

The other distinctive (F/MCb) response argued that explicit teaching of the GCs was crucial to student development academically, socially and emotionally. She utilised the GCs to develop student language skills, with one strategy being the decoration of her classroom with posters depicting specific social interactions between individuals and in groups. She used these as “discussion starters”, where students learn about how to relate and respond to others in social situations. In these classroom interactions, the teacher promoted student discussion and interaction, through “productive classroom dialogue” (Veen et al., 2017, p. 14). This approach is explored in the final section.

## Latent interview analysis using Biesta’s three functions of education

This section applies Biesta’s (2010) three functions of education as an interpretive lens to categorise participant responses and identify what participants value in education, providing a sense of “what matters” to the participant. For example, if a participant’s responses largely align with the socialisation function where the aim of education is “inserting individuals into existing ways of doing and being” (Biesta, 2010, p. 40) and developing students’ capacity to integrate into the processes and “social orders” of the school, then we are able to conclude that this function is a priority for the participant.

Biesta’s work has attracted some criticism. Jörg (2011) labels Biesta’s approach as simplistic (p. 111), claiming that Biesta fails to consider the “common prejudices, the myopia, the learner incapacities of those involved in the field and the role of outdated and blinding paradigms” (p. 111). Nonetheless, Biesta’s work has been hailed by Charles (2016) as “one of the central figures in the philosophy of education” (2016, p. 473), whilst his approach “lies in cultivating dialogical, worldly spaces and asking difficult questions that can call us into response” (Emmett, 2013, p. 2).

Biesta’s three functions are *Qualification* which “qualifies people for doing things” (Biesta, 2013, p. 128), providing the “knowledge, skills and dispositions” (p. 147) that allow one to undertake and complete a specific task or activity and is particularly associated with job qualifications and skills. Biesta asserts that this function closely relates to “economic arguments” such as the “preparation of the workforce”, highlighting the “contribution education makes to economic development and growth” (Biesta, 2010, p. 40). The *Socialisation* function, as outlined above, focuses on enculturating individuals as “members of and part of particular social, cultural and political ‘orders’” (Biesta, 2010, p. 40) and that this function can invoke school policies and procedures to enact the continued transmission of specific values and norms, evident, for example in faith-based educational institutions (Carter, 2019). The third function, *Subjectification*, centres on the “idea of uniqueness” (Biesta, 2010, p. 81) and how we differentiate ourselves from established social orders. At the heart of this function is interaction with others which provides scope for the development of different opinions, ways of thinking and rationality as the basis for “responsible responsiveness to alterity and difference” (p. 41). Some participant responses are clearly a mix of one or more of the functions. We venture here that the first two of these functions tend towards conservation of the status quo, rendering subjectification particularly important.

### Qualification function

Few responses fell into this category. This is unsurprising, given that participants are primary school teachers and provisioning their students with the knowledge and skills that will provide future employment opportunities for their students is a task for the later stages of school-based education. However, a small minority of participants cited the importance of “qualification” in the sense that it “qualifies people for doing things” (Biesta, 2013, p. 128) and in the primary school context, five participants indicated that they considered the acquisition of skills in the areas of music, drama, sport and public speaking (and attendant recognition through awards), played an important role in education. One remarked “There are those students who may not ever excel academically but excel in other areas such as sport and we need to acknowledge this in public ways” (M/MC). Another indicated, “The NAPLAN tests make my kids nervous, but I try to explain to them that they can succeed, and it will be an important milestone for them, an important achievement” (F/MCc).

### Socialisation function

Socialisation had a stronger presence in participant responses. Here, 25 participants signalled the importance of developing student attitudes and behaviours that facilitate and accelerate student “immersion” into school processes, positive peer interactions and peer groupings for different purposes. Comments from participants confirmed the importance of socialisation to a majority of participants with comments including “I need to make sure they know how to relate to each other and know how to work together” (F/LC); “One of the important things is to get them to understand how to behave and contribute in class discussions” (M/LC); and “While I want them to be relaxed in class and interact, they need to know that there are boundaries” (F/MCa). Participant comments were focused on student behaviours that gave scope for students to participate positively and productively in activities, particularly group work and discussions.

### Subjectification function

This function was identifiable through interviewees targeting the development and shaping of student attributes and personal qualities that enhance individuals’ sense of uniqueness. Subjectification centres on opening spaces for students to shape their own opinions and “test” these with other students; it is a component in the ability to thinking and rationality as a basis for “responsible responsiveness to alterity and difference” (Biesta, 2010, p. 41). This function enables students to confront difference. Whether it be in discussions, issues encountered in texts or previously unencountered ideas, subjectification requires the individual to respond, to “take a stand” on an issue, where previously half-baked, semi-formed notions and opinions are able to be expressed in a way that says “this is me” (Carter, 2019).

Fifteen participant comments invoked the subjectification function. This is perhaps unsurprising, in that research into teacher attitudes identifies teacher commitment to holistic child development (Kemp & Reupert, 2012; Pillen et al., 2013) centring on “holistic constructs of teaching that embrace the personal and social as well as the cognitive and academic” (Devine et al., 2013, p. 103). Responses representing this function included the following:

- “(We) encourage our children to explore…just like global citizens…(so) they can really value intercultural understandings…I designed this unit of work to strengthen children’s understanding of the multicultural nature of this society” (F/MC);
- “We believe our programming is actually getting them ready for the future…it’s about getting my kids ready to be critical and creative learners; be able to solve a problem with reasoning…whether they are in the classroom, at school or outside” (F/MC);
- “I use them [GCs] a lot…to get a holistic view and make sure I’m coming at the content from all angles so it can suit all the students” (F/EC);
- “I use picture books to get kids talking—discussing and sometimes debating the issues. It helps them to take a position on issues” (M/MC);
- “We’ve prioritised asking our children to respond to ideas and issues, not only to help with their literacy skills but also to make sure they are developing thinking and reasoning skills” (F/MC).
- “There’s pressure with NAPLAN and improving the kids’ results but the General capabilities allow us to work towards important goals to make our kids happy and valued individuals” (M/LC).

## Summary and discussion

As described above, curriculum studies is a site of ongoing contestation. Complexity is a constant feature and manifests itself variously, for example curriculum as “pre-active/rhetorical”, curriculum as “enacted” and implemented, the tension between a “knowledge-based” curriculum and a “competency-based” curriculum (Rasmussen et al., 2021), questions of equity and access (Pring, 2018) and as Yates (2018) notes, the place of knowledge, creativity, twenty-first century skills and so on (p. 142). The analysis of the data identified five main themes:

1. Participants ascribe value to the general capabilities;
2. Most participants do not formally integrate the general capabilities into their teaching/learning programs; they address them informally and incidentally largely through “productive talk moves” which act as conversational tools to encourage students to elaborate and clarify (Edwards-Groves, 2014, p. 1; Michaels & O’Connor, 2015, p. 334);
3. Most participants address the general capabilities through the textual study of big books, picture books and chapter books;
4. Participants value and enact a “long-term” view of education, seeing themselves equipping students for contexts beyond the classroom.
5. For most participants, the classroom implementation of the GCs acted as “embodiments of potential” (Ben-Peretz, 1990, p. 45) where student learning extended beyond the formal aims embedded in their teaching and learning programs.

We briefly elaborate each of these themes below.

1. *Participants ascribe value to the general capabilities*

The most apparent feature of participant responses in this project was the importance they ascribed to the ideas underpinning the GCs. Participants value and work towards developing and strengthening their students’ capacities for rational and reasoned thinking; providing opportunities for creativity in classroom activities; and facilitating student understanding of and behaviour in, for example, intercultural understanding. Whilst most participants implement the GCs incidentally through their teaching/learning programs, a minority deliberately integrates the GCs into programs. One participant indicated that the school’s programming template had been amended to include the GCs as a checklist, whilst another reported that the GCs had been incorporated into student reports, including student-compiled reports on their own sense of attainment.

2. *Addressing the GCs informally and incidentally*

As indicated above, most participants, through their day-to-day teaching, work towards integrating the principles of the GCs into their classroom structures and processes. This is apparent in their accounts of how they facilitate classroom discussions and interactions and is reminiscent of “dialogically orchestrated classroom talk” (Veen et al., 2017, p. 14), known variously as “productive classroom dialogue” (p. 14), “productive talk moves” (Michaels & O’Connor, 2015, p. 334) and a “dialogic learning environment” (Edwards-Groves, 2014, p. 1). This illustrates a shift from teacher-centred talk to the development of student language skills through children sharing, expanding or clarifying their ideas; listening to each other and taking each other’s ideas seriously to deepen their understanding; and building on each other’s ideas (Michaels and O’Connor, 2015). This approach to classroom dialogue was reflected in participant references to facilitating classroom talk, with the following teacher provocations emblematic of their classroom approach:

1. Can you say more about it? (broadening their ideas);
2. Who thinks they understood what was said and can put into their own words? (active listening);
3. Why do you think that? (deepening their ideas);
4. Can you add to this idea? Do you agree/disagree? Why? (thinking and building ideas together) (Michaels & O’Connor, 2015, p. 334).

Participants were adept at this approach to classroom talk and in doing so, addressed the GCs and in particular, “intercultural understanding”, “critical and creative thinking” and the “personal and social capability”.

3. *Addressing the GCs through the textual study*

The benefits of textual study as a vehicle for learning about the world can offer us “nuggets of purported wisdom” (Medway, 2010, p. 4). Moreover, “stories and literature play an important and necessary role in understanding the past and in creating the future” (McLean Davies et al., 2021, p. 1). Our participants affirmed the benefits of textual study — not only from the language and structural features of the text — but also for the opportunities for students to explore aspects related to the GCs of “ethical understanding” and intercultural understanding. The ensuing discussions and activities assisted students to think critically and creatively as well as developing their interpersonal skills. Participants argued that the knowledge and skills developed through textual study and associated activities were important for a “long-term” view of education, as outlined below.

4. *Participants value and enact a long-term view of education*

Another prominent finding was uncovered by the “latent” analysis of data. As mentioned earlier, this type of analysis seeks to “capture implicit meaning, such as ideas, meanings, concepts and assumptions which are not explicitly stated, a ‘deeper’ level of analysis” (Terry et al., 2017, p. 11). To undertake this level of analysis, we utilised Biesta’s three functions of education (Biesta, 2013) and what emerged was the dedication of participants to the holistic development of their students, despite numerous pressures such as the literacy/numeracy tests in the form of NAPLAN and associated pressures to improve student performance, as well as teacher accountability requirements. Whilst primary teacher optimism, perseverance, motivation (Mansfield et al., 2018) and the factors that sustain teachers and equip them to flourish rather than just survive in the profession are well documented (Gu & Day, 2007; Kitching et al., 2009; Sumsion, 2003), the findings here confirm the ability of participants to adopt a long-term view of their students’ education. That is, participants were dedicated to ensuring that their students were equipped with not only literacy and numeracy skills but also the self-awareness and interpersonal skills that will shape them into positive, supportive community members and help them to establish and maintain positive and fulfilling relationships. These participants see education as equipping students with the ability to “discern faulty arguments, generalisations and assertions” (Goodson & Gill, 2014, p. 42). As one participant commented, “I want them to be able to be successful at school but more importantly, be able to function effectively and positively in our multicultural society” (F/MC).

5. *Participant diverse interpretations and uses of curriculum materials*

This research project also illuminated participants’ ability to use the GCs as a “springboard” to enable beneficial student learnings, despite approximately 47% stating that they considered their understanding of the GCs to be incomplete (see question *2.1* above). Teacher agency, including how teachers interpret and implement the curriculum, has been the focus of considerable research; however, less attention has been paid to the “potential” of curriculum materials (Deng, 2011), understood as “embodiments of potential” (Ben-Peretz, 1990, p. 45). This notion, stemming from the work of Schwab (1973) who investigated the manifold ways that teachers use curriculum, was further developed by Ben-Peretz (1975, 1990) and provides a useful “lens” by drawing attention to how curriculum materials afford the opportunity for unintended student learning. Here, the curriculum materials constitute the GCs.

In using the GCs as “embodiments of potential” (Ben-Peretz, 1990, p. 45), participants interpreted the GCs in diverse ways for their classrooms: through textual study. project-based learning. discussions harnessing “productive talk moves” (Michaels & O’Connor, 2015, p. 334) and drama as a means for developing relationships and understandings. Whilst these approaches constitute deliberate teacher choices of pedagogy and resources, participants indicated that occasionally, student learning “strayed” into unintended but rich learning opportunities. This is exemplified by one specific example where one participant (M/EC) described an incident in his classroom between two students. The class had been building model clocks in small groups for several months and one student’s model was wilfully damaged by a classmate. Rather than reacting negatively to the event, the targeted student remained calm and advised the teacher “not to worry” because “working with Nicholas^2^ makes me realise some kids have it tough at home”. The participant teacher remarked that he considered this outcome as a “direct and fortunate result of class discussions about ethics and intercultural understanding” (M/EC) rather than teacher-directed instruction concerning class behaviour.

## Conclusions and recommendations

Most of the participants value the GCs. However, only a small minority of them are required to integrate the GCs into their teaching/learning programs. Numerous participants in both the online survey and the interviews argued for greater professional learning provided by the relevant school sector to deepen the knowledge and possibilities for classroom integration of the GCs.

Curriculum authorities and the school sectors need to consider the status and role of the GCs in their respective educational jurisdictions and key questions need addressing:

- Should teachers be required to formally address the GCs in teaching/learning programs?
- If the GCs are deemed important, how can they be integrated effectively into teaching/learning programs without overloading the classroom teacher?
- What resources will support teachers in addressing the GCs?

We add a provocation here, although we are loath to add to teachers’ burdens by insisting on GC programming. What if we were to adopt a GC-led curriculum, in which the capabilities supersede or replace subject-related outcomes? We propose the GCs of C and CT, P and SC, EU and IU as “higher-order” capabilities. Literacy, numeracy and ICT can be seen as tools for attaining these.

The answers to these questions can be shaped by broadly based discussions with teachers, school leaders and curriculum designers, as well as this project’s participants. Their passion and commitment to their teaching is undeniable, and will flourish from further support and consultation.

## Footnotes

1 ‘F’ identifies female participants. ‘M’ identifies male participants. ‘EC’ denotes early career teacher 0–5 years. 6 ‘MC’ denotes a mid‐career teacher with 6–10 years of teaching experience. ‘LC’ denotes a later career teacher with ten or more years of teaching experience. Superscript ‘a’, ‘b,’ etc. denote multiple participants in the same category.

2 A pseudonym.
